# Light‐Controlled Magnetic Properties: An Energy‐Efficient Opto‐Mechanical Control over Magnetic Films by Liquid Crystalline Networks

**DOI:** 10.1002/advs.202408273

**Published:** 2024-10-07

**Authors:** Gabriele Barrera, Daniele Martella, Federica Celegato, Neri Fuochi, Marco Coïsson, Camilla Parmeggiani, Diederik S. Wiersma, Paola Tiberto

**Affiliations:** ^1^ Istituto Nazionale di Ricerca Metrologica (INRiM) Strada delle Cacce 91 Torino 10135 Italy; ^2^ European Laboratory for Non Linear Spectroscopy (LENS) Via N. Carrara 1 Sesto Fiorentino Firenze 50019 Italy; ^3^ Department of Chemistry “Ugo Schiff” University of Florence Via N. Carrara 3–13 Sesto Fiorentino 50019 Italy; ^4^ Department of Physics and Astronomy University of Florence via G. Sansone 1 Sesto Fiorentino 50019 Italy

**Keywords:** liquid crystalline networks, magnetostrictive thin film, photoresponsive actuators, remote control, tunable magnetic properties

## Abstract

Magnetostrictive materials are essential components in sensors, actuators, and energy‐storage devices due to their ability to convert mechanical stress into changes in magnetic properties and vice‐versa. However, their operation typically requires physical contact to apply stress or relies on magnetic field sources to control magnetic properties. This poses significant limitations to devices miniaturization and their integration into contactless technologies. This work reports on an approach that overcomes these limitations by using light to transfer mechanical stress to a magnetostrictive device, thereby achieving non‐contact and reversible opto‐mechanical control of its magnetic and electrical properties. The proposed solution combines a magnetostrictive Fe_70_Ga_30_ thin film with a photo‐responsive Liquid Crystalline Network (LCN). Magnetic properties are modulated by changing the light wavelength and illumination time. Remarkably, the stable shape change of the LCN induced by ultraviolet (UV) light leads to the retention of magnetic properties even after the light is switched off, resulting in a magnetic memory effect with an energy consumption advantage over the use of conventional magnetic field applicators. The memory effect is erased by visible light, which releases the mechanical stress in the photoresponsive layer. Therefore, this new composite material creates a fully reconfigurable magnetic system controlled by light.

## Graphical abstract

Control on the magnetic properties and resistance of a FeGa thin film is obtained by coupling it with a photoresponsive Liquid Crystalline Network. The photo‐induced stress of the polymeric substrate is able to efficiently modulate the properties of the metallic layer in a reversible way.

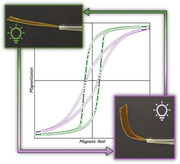

## 1 Introduction

The synergic combination of functional materials whose properties can be tuned by external stimuli such as magnetic fields, temperature, mechanical stress, and light, has enabled the realization of multifunctional smart composite materials with breakthroughs in various fields of application, spanning from actuating systems to sensors and photonic devices.^[^ ^1^, ^2^, ^3^, ^4^, ^5^ ^]^

In this context, magnetostrictive materials enable a unique bi‐directional coupling between magnetic and mechanical energy. These materials either respond to an applied magnetic field by exhibiting deformations or transduce external mechanical stresses into internal magnetic anisotropy, resulting in a measurable change in magnetic properties. The magnetostrictive effect has been widely employed for the realization of magnetic field sensors, biological and tactile sensors,^[^ ^6^, ^7^, ^8^ ^]^ actuators,^[^ ^9^, ^10^ ^]^ transducers in ultrasonic devices,^[^ ^11^ ^]^ and energy harvesters.^[^ ^12^, ^13^ ^]^

The integration of magnetostrictive thin‐films with smart substrates capable of finely and locally controlling stress is currently being investigated. In that context, polymer substrates integrated with spin‐valve structures composed of magnetostrictive layers have been exploited to achieve a strain‐controlled giant magnetoresistance effect.^[^ ^14^, ^15^ ^]^ Additionally, piezoelectric substrates have been coupled with magnetostrictive thin films to create artificial magnetoelectric composite heterostructures.^[^ ^16^, ^17^, ^18^, ^19^ ^]^ Recently, silk fiber substrates coated with a magnetostrictive metal layer provided an excellent combination of silk's natural mechanical properties with stress‐sensitive magnetic properties and strain‐dependent electrical conductivity.^[^ ^20^ ^]^

The current research challenge involves activating functional substrates with reliable and peculiar external stimuli enabling the reconfiguration and fine‐tuning of the magnetic properties of a magnetostrictive thin film point by point in the same device. This would reduce the use of bulky magnets or coils to tune magnetic response, which not only takes up significant space but also leads to substantial energy consumption. This issue is particularly pertinent considering the prominent trend toward miniaturization in magneto‐electronics.^[^ ^21^, ^22^, ^23^ ^]^ Consequently, there is an urgent need for alternatives that do not rely on magnetic components for switching and modulating magnetism.

A possible solution should be the use of light irradiation as a contactless reconfiguration stimulus, which enables remote control of mechanical stress from a photoresponsive substrate to a magnetostrictive layer. Following this direction, a simple physical realization of a smart composite material, whose magnetic properties are modulated reversibly by light, is presented in this study. In particular, a magnetostrictive thin film is combined with a photoresponsive polymeric actuator allowing for wireless, non‐contact control over the mechanical strain produced inside the material when irradiated with an appropriate wavelength.

To demonstrate the concept an actuator based on a Liquid Crystalline Network (LCN) containing an azobenzene crosslinker has been realized.^[^ ^24^ ^]^ Indeed, low molecular weight liquid crystals have been used to prepare a variety of functional materials, from ordered self‐assembly of nanostructures,^[^ ^25^, ^26^ ^]^ to responsive polymers (as exploited here). On the other hand, azobenzene is a photoswitch that has been previously used to trigger variations of shape,^[^ ^27^, ^28^, ^29^ ^]^ adhesion,^[^ ^30^, ^31^ ^]^ wettability,^[^ ^32^ ^]^ and many other macroscopic properties of materials.

LCNs are materials where mesogenic molecules are chemically crosslinked to polymeric networks. Their most peculiar behavior is their reversible shape‐changing capability and sensitivity to external stimuli.^[^ ^33^, ^34^ ^]^ For example, during heating, the molecular order is gradually lowered obtaining a progressive contraction along the alignment direction and expansions in the perpendicular ones. The materials recover their original shape on cooling.^[^ ^35^, ^36^ ^]^ In azobenzene doped LCN, the liquid crystalline order can be modified by irradiation which causes the dye isomerization from the *trans* to *cis* state^[^ ^37^, ^38^ ^]^ hereby inducing a phase transition (or a partial disordering of the aligned mesogen)^[^ ^27^, ^39^ ^]^ with a consequent macroscopic deformation. These materials have been extensively studied as artificial muscles for the realization of light activated robotics systems^[^ ^40^, ^41^, ^42^ ^]^ and other miniaturized responsive devices.^[^ ^43^, ^44^ ^]^

So far, the combination of LCNs with magnetic materials has involved the integration of magnetic micro/nanoparticles^[^ ^45^ ^]^ for a remote magneto‐responsive actuation^[^ ^46^, ^47^ ^]^ (e.g., by heat dissipation under alternating current magnetic fields).^[^ ^48^, ^49^ ^]^ LCNs have been also used to orient the nanoparticles, thus allowing a control over the magnetic properties under mechanical stretching (passing through polydomain to homogeneous alignments).^[^ ^50^, ^51^, ^52^ ^]^ On the other hand, to the best of our knowledge, their use as mechanical actuators for the control over magnetostrictive thin films has never been reported.

Therefore, this research activity has been dedicated to the integration of a magnetostrictive Fe_70_Ga_30_ thin film^[^ ^53^ ^]^ with a LCN one exploring the possibility of fine‐tuning the magnetic properties without the need for a magnetic field, but only through light irradiation. Specifically, irradiation with ultraviolet (UV) light induces the photo‐actuation of the LCN substrate transferring mechanical stress to the FeGa thin film. Exploiting this mechanism, magnetic parameters (including magnetization remanence, coercive field, and magnetic susceptibility) can be modulated as a function of the UV illumination time, and are maintained constant after switching off the light. The emergence of magnetic anisotropy after irradiation is also demonstrated in dependence of the liquid crystalline alignment of the underlying layer. In addition, irradiation with visible light causes a gradual release of stress in the FeGa thin films and a consequent recovery of their pristine magnetic properties. The same reversible tuning is observed by anisotropic magnetoresistance measurements where the stress induced by light irradiation is exploited to adjust and set the resistance value of the FeGa thin film.

This work aims to pave the way for advanced and innovative contactless technologies based on the control of the magnetic properties in thin films. The proposed opto‐mechanical action addresses the urgent demand for alternative tuning mechanisms for switching and modulating magnetism that does not rely on bulky and heavy magnetic components. This approach has the potential to promote safe energy consumption and enhance miniaturization in the field of magneto‐electronics.

## 2 Results and Discussion

### 2.1 Material Preparation and Photoresponsive Actuation

**Figure 1:**
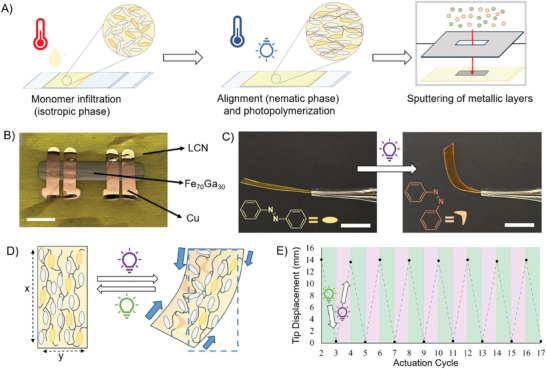
Preparation of FeGa/LCNs and photoresponsive shape‐change. A) Scheme of the steps for the material fabrication. B) Picture of a final FeGa/LCN sample. Scale bar: 5 mm. C) Pictures of a LCN stripe before (right) and after (left) irradiation with UV light from the top. Scale bars: 1 cm. D) Scheme of the photoresponsive behavior mechanism during the irradiation with UV or visible light. E) Example of the photoactuation reversibility. The graph reports the tip displacement of a FeGa/LCN stripe after irradiation with UV and visible light respectively. Using different LED sources, the stripe first bent toward the light (during UV irradiation) and then recovered the original position (with green light).

Responsive actuators have been obtained by photopolymerization of a mixture of acrylate‐based reactive mesogens.^[^ ^54^ ^]^ The structure of the monomers is reported in Figure S1 (Supporting Information) and the mesomorphic properties of the mixture have been studied by Differential Scanning Calorimetry (DSC) and Polarized Optical Microscopy (POM) as reported in Figure S2 (Supporting Information). Such analyses demonstrate the presence of a nematic phase both during the heating and the cooling stage. The steps for material fabrication are briefly summarized in **Figure** **1**A. First, the mixture is melted in the isotropic phase and infiltrated by capillarity in a liquid crystalline cell, treated to obtain a homogeneous planar alignment. The sample is cooled down in the nematic phase and photopolymerized by a blue LED (450 nm). All the polymeric films presented a monodomain homogeneous alignment with the mesogenic molecules that are aligned parallel to the film surface along a common direction (nematic director), as checked by POM (Figure S3, Supporting Information).

The LCN layer is subsequently used as a substrate for a 30 nm thick magnetic Fe_70_Ga_30_ film deposition using the sputtering technique with an appropriate mask to select the metal layer shape. To study the magnetoresistive properties of the FeGa/LCN sample, four copper electrical contacts are sputtered on top of the magnetic thin film. An optical image of the final composite material is shown in Figure 1B while two pictures taken by a Scanning Electron Microscope (SEM) of its cross section are reported in Figure S4 (Supporting Information).

An example of the light‐responsive behavior of a LCN stripe under UV illumination (LED lamp, 385 nm) is reported in Figure 1C. The initial (almost flat) polymeric film undergoes a bending toward the light source when irradiated (from the top)^[^ ^55^, ^56^ ^]^ and retains the bent shape after switching off the light (due to the stability of the *cis* isomer). The mechanism for the material shape‐change is schematically depicted in Figure 1D. The dye isomerization triggers a progressive disordering of the molecular order to obtain a contraction along the alignment direction and the expansion in the perpendicular ones (labeled *x*‐ and *y*‐direction, respectively).^[^ ^57^, ^58^ ^]^ The high absorbance of the sample in the UV region causes a gradient light penetration along the thickness of the material, with non‐homogeneous shrinking across the sample, giving rise to a macroscopic bending motion.^[^ ^59^, ^60^ ^]^ The material shape‐change is reversible by irradiation with a green LED lamp (505 nm) that induces the opposite molecular reaction (*cis‐trans* isomerization) and restores the original nematic order. A peculiar behavior of the chosen alignment is that the bending occurs toward the irradiated side (see Figure S5, Supporting Information for an example of bending in different directions by the same LCN stripe).

Very interestingly, the presence of a FeGa metallic skin on one side of the actuator does not hamper the photoinduced bending as demonstrated in Movie S1 (Supporting Information) (showing both bending under UV and unbending with visible light). The light source faces the side of the LCN opposite to where the FeGa thin film was deposited, resulting in a homogeneous deformation of the FeGa/LCN samples similar to that observed for the pristine LCN reported in Figure 1C; consequently, this setup configuration will be used for all subsequent characterizations.

On the other hand, if the irradiation is performed on the same side of the FeGa layer, the LCN is not able to bend efficiently and homogeneously (since the metallic layer hinders the absorption by the polymeric film behind) thus limiting shape‐change with respect to the pristine LCN.

The reversibility of the photoinduced bending for the FeGa/LCN is demonstrated in Figure 1E, reporting the tip displacement (measured at the end of the strip by optical images) for a FeGa/LCN actuator after several cycles of UV and green light irradiation. As the number of cycles progresses, there is no evidence of any degradation of the LCN deflection properties. For a similar polymer composition, it has already been demonstrated by force measurements that this shape‐change functionality is preserved over thousands of irradiation cycles.^[^ ^61^ ^]^ Additional exemplificative pictures to demonstrate the shape‐changing reversibility are also reported in Figure S6 (Supporting Information).

### 2.2 Opto‐Mechanical Modulation on Magnetic Properties

Room temperature hysteresis loops of the FeGa/LCN sample are measured by applying a magnetic field *H* in the film plane along both directions (already labeled *x* and *y*, referring to the directions parallel or perpendicular to the LC alignment, respectively). The FeGa/LCN sample is fixed on a glass plate to prevent macroscopic bending of the photoresponsive substrate ensuring that the plane of the FeGa thin film is always parallel to the applied magnetic field during the measurements. Nevertheless, this sample mounting does not hinder the photoisomerization of the dye contained in the polymeric structure.

The *M*(*H*) curves, corresponding to the FeGa/LCN sample in the initial state (i.e., before any illumination), are shown in Figure S7 (Supporting Information). The two curves appear practically overlapping with a slightly higher coercive field and remanence magnetization along the *y*‐direction. These features indicate an almost isotropic magnetic behavior, with a slight preference to magnetize the sample along the *y*‐direction. In this initial stage, no stress is applied to the FeGa thin film by the underlying polymer matrix; consequently, the corresponding *M*(*H*) curves are used as a reference for the further magnetic characterizations.

**Figure 2:**
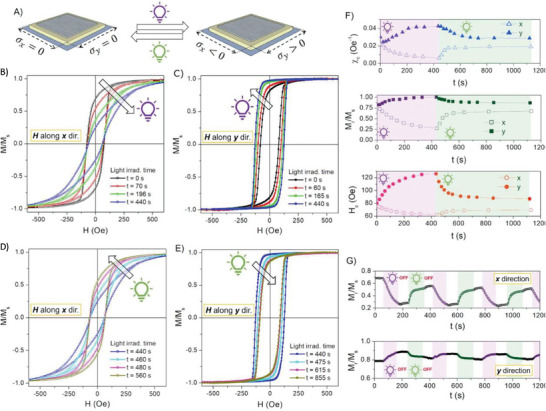
Light‐induced modulation of the FeGa/LCN magnetic properties. A) Sketch of the opto‐mechanical action from irradiated LCN substrate to magnetostrictive FeGa thin film. B,C) Room‐temperature normalized hysteresis loops after UV light irradiation for selected time with the magnetic field *H* applies along the *x* and *y* direction, respectively. D,E) Normalized hysteresis loops at room temperature after green light irradiation for selected time (following the UV irradiation reported in panels B and C)) with the magnetic field *H* applies along the *x* and *y* directions, respectively. F) Evolution of normalized saturation magnetization (*M_r_/M_s_*), coercive field (*H_c_*), and magnetic susceptibility (*X_Hc_*) evaluated along *x* and *y* directions as a function of progressive UV and green irradiation time. G) Modulation of *M_r_/M_s_* value at *H* = 0 Oe along *x* and *y* direction by subsequent application of UV and green light. Color code in (F) and (G): the area shaded in purple, green, or white indicates irradiation with UV or green light or both lamps off, respectively.

As stated in the previous section, UV irradiation on the LCN substrate activates its deformation which is transferred as a complex mechanical shear stress to the FeGa thin film (σ_x,y_), as sketched in **Figure** **2**A. To maximize stress transfer, the light source is positioned directly on the side of the sample opposite to the FeGa thin film deposition. This applied stress results in a compressive and tensile strain of the magnetic thin film along the *x‐* and *y‐*direction, respectively. These opto‐mechanical actions significantly affect the magnetic properties of the FeGa thin film, as shown in Figure 2, owing to its considerable magnetostrictive character.^[^ ^53^, ^62^ ^]^ A comprehensive discussion of the opto‐mechanical mechanism together with a detailed sketch (Figure S8, Supporting Information) can be found in the Supporting Information.

By measuring the magnetic properties along the compression direction (*H* along the *x* direction), a progressive change in the shape of the hysteresis loops is observed with increasing UV irradiation time (Figure 2B) up to *t* = 440 s. In particular, the reversal of magnetization occurs over an increasingly wide magnetic field range. Consequently, the magnetic field intensity necessary to overcome the effective magnetic anisotropy energy present in the FeGa thin film, and thus reach magnetic saturation, becomes progressively higher.

Conversely, the opposite behavior is observed measuring the *M(H)* curves along the tensile direction (*H* along the *y* direction) as a function of UV light irradiation time (Figure 2C) up to *t* = 440 s. The reversal of magnetization becomes progressively steeper and occurs in an increasingly narrow magnetic field range.

A detailed evolution of the magnetic parameters such as normalized magnetization remanence (*M_r_/M_s_*), coercive field (*H_c_*) and magnetic susceptibility (*X_Hc_*) evaluated from the *M*(*H*) curves in the *x*‐ and *y*‐directions are reported in Figure 2F as a function of UV light irradiation time (up to 440 s). All parameters show a monotonic approach to a constant value as the irradiation time increases. However, the trend is opposite as a function of the direction along which the magnetic parameters are measured: a decrease along the compressive *x*‐direction and an increase along the tensile *y*‐direction.

In particular, the magnetic susceptibility shows a symmetrical deviation of about 70% from the initial values in both directions. Instead, the normalized magnetization remanence tends toward its upper limit of *M_r_/M_s_* = 1 along the *y*‐direction, while it shows a greater decrease along the *x*‐direction reaching a value almost three times smaller than the initial one. The coercive field mainly favors the *y*‐direction with an increase of more than 50% from the initial value.

This ability to control the magnetic behavior of the thin film through the opto‐mechanical effect of the LCN is closely related to the significant positive magnetostrictive properties of FeGa alloy.^[^ ^53^, ^62^ ^]^

In the absence of induced mechanical strain in the FeGa thin film (at irradiation *t* = 0 s), the shape of the *M*(*H*) curve is mainly governed by the magnetocrystalline and shape anisotropies.^[^ ^63^ ^]^ These anisotropies are distributed almost isotropically in the film plane, as confirmed by the near‐overlap of the hysteresis loops measured along the *x* and *y*‐directions for the as‐deposited thin film (see Figure S7, Supporting Information). When the mechanical strain is induced by the LCN substrate in the FeGa layer during UV irradiation, an uniaxial stress anisotropy is generated with the magnetic easy axis along the *y*‐direction (i.e., the direction of the tensile strain),^[^ ^53^, ^63^ ^]^ leading to anisotropic magnetization behavior. The uniaxial stress anisotropy becomes stronger as the UV illumination time increases.

When measuring magnetic properties along the *x* direction (i.e., *H* along the hard axis of the magnetic stress anisotropy), the induced stress anisotropy competes with the applied magnetic field in driving the magnetic moments. Therefore, the gradual increase in the intensity of the stress anisotropy as a function of the duration of the UV irradiation leads to a gradual increase in the magnetic field strength required to reach magnetic saturation (Figure 2B). As a result, a decrease in the values of *M_r_/M_s_*, *H_c_*, and *X_Hc_* as a function of the UV irradiation time is observed (Figure 2F).

In contrast, when studying the magnetic properties along the *y*‐direction (i.e., *H* along the stress anisotropy easy axis), the applied magnetic field and stress anisotropy cooperate in driving the magnetic moments. Therefore, by increasing the intensity of the stress anisotropy through the UV irradiation time, the magnetization is increasingly favored to remain along the magnetic anisotropy easy‐direction axis, showing a steeper inversion process (Figure 2C). Consequently, an increase in *M_r_/M_s_*, *H_c_*, and *X_Hc_* values is observed as a function of the UV irradiation time (Figure 2F).

The release of the stress‐induced in the FeGa thin film during UV irradiation is obtained by green light (Figure 2A), which induces the opposite molecular reaction (cis‐trans isomerization) with respect to UV light and restores the original nematic order. As a result, the intensity of the uniaxial stress anisotropy decreases and the magnetic properties of the FeGa/LCN sample tend to progressively recover to their initial state (*t* = 0 s). In fact, the shape of the *M(H)* curve induced after 440 seconds of UV irradiation shifts progressively as a function of the green irradiation time until the shape of the initial stage is partially recovered, as shown in Figure 2D,E.

Similarly, it occurs for the magnetic properties *M_r_/M_s_*, *H_c_*, and *X_Hc_*, as shown in Figure 2F. Short time intervals of green light induce a prompt variation in the magnetic parameters which is in opposition to the effects observed under UV light. Following this initial response, after approximately 640 seconds, the magnetic properties approach a constant value, resulting in a partial recovery of the initial magnetic state (*t* = 0 s). In particular, the final magnetic values are found to be within 10% of the initial ones for all parameters.

Furthermore, this competitive action of UV or green light irradiation in modulating the strain in FeGa thin film allows its magnetic properties to be adjusted over a wide range of values without the need for magnetic fields.

As an example, in Figure 2G, the normalized magnetization remanence is modulated by subsequent application of UV and green light. The UV‐light irradiation linearly adjusts the *M_r_/M_s_* value toward a lower or higher value for the *x*‐ and *y*‐directions, respectively, compared to the initial state. It is important to note that the *M_r_/M_s_* value set by the UV irradiation is maintained even after the light is turned off, demonstrating a magnetic memory effect. For both directions, the green‐light is able to tune the *M_r_/M_s_* value in the opposite trend (higher or lower value for the *x*‐ and *y*‐directions, respectively). A few cycles of UV/green irradiation are carried out and, except for the first cycle where a slight reduction in the magnetic signal is observed, probably due to the release of peculiar stresses generated at the material interface during the sputtering deposition, the subsequent cycles result in a satisfactory reproducibility of the magnetic signal modulation.

Moreover, the light‐induced modulation of the *M_r_/M_s_* value can be adjusted in intensity not only through an appropriate irradiation time but also by synergically applying a constant magnetic field in the sample plane (e.g., via an electro‐magnet or a permanent magnet). As shown in Figure S9 (Supporting Information), increasing the magnitude of the constant magnetic field results in a reduction of the light‐induced modulation effect. This effect occurs due to the competitive action of the magnetic field and the uniaxial stress anisotropy on magnetic moments. At *H* = 600 Oe, the effect of the magnetic field almost overcomes the stress anisotropy energy while a negligible effect of light on *M_r_/M_s_* value is observed. This cooperative action extends the multi‐response aspects of the FeGa/LCN sample to model its magnetic properties.

In the present study, the irradiation time was used as the key parameter to modulate the light dose to the FeGa/LCN sample, thus controlling the magnetic properties. Note that an equivalent effect can be achieved by using the light power as a control parameter; an example is shown in Figure S10 (Supporting Information).

### 2.3 Opto‐Mechanical Modulation on Magnetoresistive Properties

The light‐induced modulation of magnetization in FeGa/LCN sample was also studied by anisotropic magnetoresistance measurements, in which the resistance of the FeGa thin film (*R*) varies as a function of the angle between the direction of magnetization and the direction of the electric current (*I*) flowing in the magnetic layer.^[^ ^62^, ^64^ ^]^

**Figure 3:**
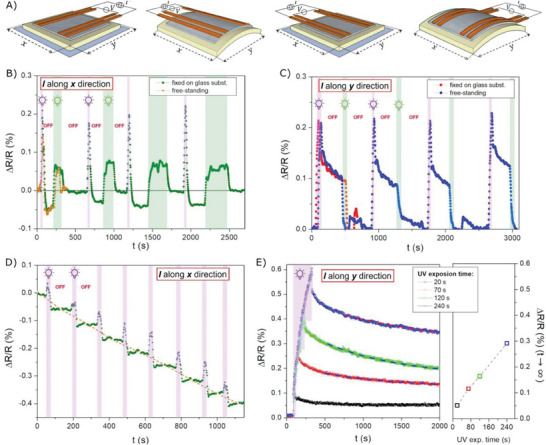
Light‐induced modulation of the FeGa/LCN magnetoresistance properties. A) Sketch of the opto‐mechanical action from irradiated LNC substrate to magnetostrictive FeGa thin film leading to a macroscopical bending; UV and green light‐induced modulation of ∆*R/R* value of FeGa/LCN sample both fixed and free to bend by flowing electric current along, B) the *x*‐direction, and C) along the *y*‐direction. D) Response of *∆R/R* value to successive UV irradiations of ∆*t* = 20 s. E) Response of *∆R/R* value at different UV exposure times. Color code: the area shaded in purple, green, or white indicates irradiation with UV or green light or both lamps off, respectively.

Magnetoresistance measurements were performed on the FeGa/LCN sample both fixed on a glass layer and free to bend by flowing electric current in both the in‐plane directions, as schematized in **Figure** **3**A. Specifically, the *∆R/R* value is recorded for an interval of time during which the UV and green light were properly turned on and off.

Figure 3 shows that an increase in resistance occurs during light irradiation for both current configurations (*I* parallel to the *x* or *y*‐directions) and light wavelengths, although the effect is more significant when using the UV lamp rather than the green lamp. This increase is mainly due to a non‐negligible thermal effect in the FeGa sample induced by the light, as described in the Supplementary Information and shown in Figure S11 (Supporting Information). Therefore, the following description has been focused on the Δ*R/R* value when the lamps are off. After thermal relaxation following the lamp switch off, the ∆*R/R* value results in a different value than the initial one. This *∆R/R* modulation is strictly related to the shear strain induced in the FeGa thin film by the photoinduced isomerization of the dye. Indeed, it has been proved that a FeGa/LCN sample without photosensitive molecules in the polymer matrix exhibits a *∆R/R*(t) curve that returns perfectly to its initial value after the light (both UV and green) has been switched off and the thermal relaxation has ended, as shown in Figure S12 (Supporting Information).

Figure 3B reports the results obtained by applying the electrical current along the *x‐*direction (i.e., the direction along which FeGa undergoes compressive deformation due to UV irradiation) for the FeGa/LCN sample in the fixed state (green curve). After UV lamp irradiation (Δ*t* = 20 s), a significant reduction in the *∆R/R* parameter from its initial value (at *t* = 0 s) is evident. Also in this case, this value remains constant even after the lamp is turned off, confirming an interesting magnetic memory effect. Subsequently, *∆R/R* is restored to its initial value by irradiation with green light (Δ*t* = 120 s). This experimental sequence is repeated four times, demonstrating the repeatability of the observed light‐induced modulation of resistance.

Conversely, when applying the electric current along the *y* direction (i.e., the direction along which FeGa undergoes tensile deformation due to UV irradiation), a significant increase in the *∆R/R* parameter from the initial value (at *t* = 0 s) is recorded after UV irradiation, as shown in Figure 3C. In particular, the intensity of the *∆R/R* modulation in this configuration is more than double that of the case with*I*parallel to the *x* direction. However, the *∆R/R* value does not remain constant at the value reached immediately after turning off the UV lamp, but slowly decreases toward a lower value, which is still higher than the value at *t* = 0 s. To return the system to its initial state (*∆R/R* = 0), irradiation with green light is needed. The repeatability test of resistance behavior was again performed for the fixed sample, and the result is fully satisfactory.

The long‐term functionality of the FeGa/LCN device was evaluated by comparing measurements of the *∆R/R* modulation performed two months apart. The same samples are used for all measurements and both *I* configurations are investigated. The experimental curves, shown in Figure S13 (Supporting Information), indicate that the ability to modulate the magnetoresistive properties of the FeGa thin film by light irradiation is effectively preserved over this time interval.

Interestingly, when the FeGa/LCN sample is free to bend, the stress induced by the curvature of the substrate^[^ ^65^ ^]^ due to the UV light irradiation does not further affect the intensity of *∆R/R* variation in both current configurations. In fact, as shown in Figure 3B,C, the curves (orange line for *I* parallel to the *x*‐direction and red line for *I* parallel to the *y*‐direction) are perfectly superimposed to the ones for the fixed samples, where only the shear stress at the materials interface occurs. It is important to highlight that in the present case, the stress generated by substrate bending acts in opposition to shear stress. Indeed, along the *x*‐direction, the first results in a tensile force on the FeGa thin film whereas the second exerts a compressive one. Conversely, along the *y*‐direction. Therefore, this evidence suggests that the opto‐mechanical effect is preferentially guided by a high strain at the interface between the two materials that overcome the lower strain induced by the macroscopic deformation of the LCN.

As a matter of fact, the light‐induced modulation of the *∆R/R* value in fixed and free samples is closely related to the control of uniaxial stress anisotropy in the FeGa thin film, which is provided by the shear stress induced by the molecular rearrangement into the LCN substrate under irradiation. The onset of the stress anisotropy under UV light favors the reorganization of the FeGa magnetic moments into an average magnetization that is more oriented along its easy axis (i.e., along the tensile *y* direction) and away from its magnetic anisotropy hard axis (i.e., the compressive *x*‐direction)^[^ ^63^ ^]^ compared to the initial state (*t* = 0 s). This behavior has an opposite effect on the *∆R/R* value depending on the direction of the electric current used for the magnetoresistance measurements. When measuring along the *y*‐direction, magnetization and current tend to align parallel, leading to an increase in the *∆R/R* value (Figure 3C). Conversely, when measuring along the *x*‐direction, they misalign, resulting in a decrease in the ∆*R/R* value (Figure 3B).

Figure 3D shows the response of the *∆R/R* value to successive UV irradiations of ∆*t* = 20 s; the electrical current is applied along the *x*‐direction. A reduction in the resistance value is observed after each irradiation time, which is maintained until the next irradiation. The decrease in the *∆R/R* value is consistent, resulting in a staircase‐shaped curve with an overall decrease of approximately 0.4%.

Figure 3E shows the response of the *∆R/R* value at different exposure times (∆*t* = 20–240 s) to UV light; the electric current is applied along the *y*‐direction. As expected, the longer the UV irradiation time, the greater the increase in the *∆R/R* value. Each curve, after switching off the light, shows a slow decrease toward an asymptotic value (similarly to the case reported in Figure 3C) that is evaluated by a fitting with an exponential law (dashed line). The asymptotic values, reported in the right framework of the Figure 3E, are linearly related to the UV exposure time providing a simple and non‐contact way to adjust and set the resistance value of the FeGa thin film.

The intensity of the *∆R/R* variation can therefore be modulated not only by the irradiation time but also by applying a constant magnetic field in the sample plane, as shown in Figure S14 (Supporting Information). This effect is similar to the previously described *M_r_/M_s_* behavior. Increasing the magnitude of the constant magnetic field reduces the light‐induced ∆*R/R* modulation effect.

This demonstrates once again that light irradiation and the magnetic field can be used as co‐operating tools to fine‐tune the magnetoresistance properties of the magnetostrictive thin film.

## 3 Conclusion

In summary, this research activity has successfully demonstrated how the combination of a photoresponsive polymeric actuator with a magnetostrictive Fe_70_Ga_30_ thin film leads to a smart composite system, whose shape and magnetic properties can be finely tuned with light. Specifically, the irradiation of the photoresponsive layer with UV or green light promotes molecular reconfiguration, generating mechanical energy at the interface with the magnetostrictive FeGa thin film. This results in strain of the magnetic layer, affecting its magnetic behavior through the formation of uniaxial stress magnetic anisotropy. The appropriate selection of the light wavelength and irradiation time enabled the magnetic properties of the smart composite material to be adjusted over a wide range of values, reversibly and on demand without the need for an external magnetic field.

These multifunctional properties pave the way for the development of advanced devices based on the magnetostrictive effect, in which the modulation of magnetic properties is triggered by a non‐contact activated mechanical stress.

Interestingly, the optical mechanism chosen for the actuator also results in a magnetic memory effect, and the magnetic properties set by light are fixed even after the irradiation source is switched off until a second wavelength is used to restore the initial state.

The unprecedented possibility to both tune the magnetic properties classically, by magnetic field, and remotely by light, combined with the magnetic memory effect and the intrinsic versatility of LCN (in terms of polymeric composition and dye to be used, resulting in the light source to be used for their activation) allows for the development of a range of new smart materials to be easily integrated into more complex and miniaturized systems.

As a result, these opto‐mechanical responsive materials will advance MEMS and NEMS technology based on controlling the magnetic properties of thin films. In fact, the heavy and bulky permanent magnets or coils currently used to control magnetic properties can be replaced by light sources, leading to a reduction in manufacturing processes and, consequently, a reduction in device complexity, cost, and energy consumption. These advanced magneto‐electrical devices will find use in several cutting‐edge applications such as soft robotics, magnetic actuators and sensors, reversible magnetic memories, and physical unclonable functions.

## 4 Experimental Section

### Materials

C6BP (4‐Methoxybenzoic acid 4‐(6‐acryloyloxyhexyloxy) phenyl ester), RM82 (1,4‐Bis‐[4‐(6acryloyloxyhexyloxy) benzoyloxy]‐2‐methylbenzene) were purchased from Synthon Chemicals. Irgacure 819 was purchased from Sigma‐Aldrich. The azobenzene crossliker has been synthesized as reported in Figure S15 (Supporting Information). Polymeric network preparation is described in detail in the Supporting Information.

A 30 nm thick Fe_70_Ga_30_ thin film was deposited on an LCN layer substrate using the RF‐sputtering deposition technique. The deposition parameters were kept constant for all samples, with a base pressure of 1 × 10^−7^ mbar, a fixed target power density of 50 W, and an Ar gas pressure of 1 × 10^−2^ mbar. The deposition time was set to 250 s, based on the deposition rate of FeGa (1.2 Å s^−1^), which was previously experimentally evaluated by atomic force microscopy (AFM) measurement on a calibration sample.

The thin film composition was checked by Energy‐Dispersive X‐Ray Spectroscopy (EDS) analysis.

### Light Actuation

LCN films were irradiated by UV (ThorLabs M385CP1‐C4, λ = 385 nm, I: 12.2 mW cm^−2^) and green (ThorLabs M505L2‐C4, λ = 505 nm, I: 6.2 mW cm^−2^) LED lamps. Shape‐changing behavior was analyzed by optical images taken during and after the illumination stage. Image analysis was performed using ImageJ software (Rasband, W.S., ImageJ, U. S. National Institutes of Health, Bethesda, Maryland, USA, https://imagej.net/ij/, 1997–2018).

### Magnetic Measurements

Room‐temperature hysteresis loops *M*(*H*) were measured by a highly sensitive alternating field magnetometer (AGFM, Princeton Measurements Corporation) operating in the magnetic field range ± 18 kOe. The magnetic field was applied along both the in‐plane directions (referred to as *x* and *y*) of the FeGa thin film. The FeGa/LCN sample was affixed to the sample‐holder with the LCN layer facing the light source. Prior to measuring the hysteresis loop, the sample was exposed to UV (or green) light for the specific duration. Bending of the sample was prevented by fixing it on a glass layer, thus ensuring that the magnetic field is always applied in the plane of the FeGa thin film.

The magnetic parameters such as normalized magnetization remanence (*M_r_/M_s_*), coercive field (*H_c_*), and magnetic susceptibility (*X_Hc_*) were evaluated from the *M(H)* curves.

As a preliminary step, experimental tests were carried out to prove that the magnetic field is not able to control the orientation of mesogen in the LCN polymers and therefore provide shape‐change of the substrate with undesirable effects on magnetic modulation; see Figure S16 (Supporting Information).

### Magnetoresistance Measurements

Resistance (*R*) measurements at room temperature on the FaGa/LCN samples were performed using a standard four‐contact technique: the two external contacts were used to drive the DC drive current through the FeGa thin film, while the two inner ones were used to measure the voltage signal. A signal generator (Sourcemeter 2400 Keithley) was exploited to generate the DC current at a constant intensity of 50 × 10^−6^ A, while a digital multimeter (Nanovoltmeter 2182 Keithley) was used to measure the voltage signal.

The four contacts were made of copper by sputtering deposition technique (using the same deposition parameters as the FeGa layer), and are oriented so that the magnetoresistance behavior is measured along both directions (referred as *x* and *y*) in the plane of the FeGa thin film.

The experimental setup was equipped with a fan above the FaGa/LCN sample to improve heat dissipation and reduce the heat‐induced resistance variation. During the measurement of *R*, UV (or green) illumination of the LCN layer was performed. The relative variation ∆*R/R* = (*R*(t) – *R*(t = 0))/*R*(t = 0) × 100 was calculated and reported as a function of time.

Before each measurement, the FaGa/LCN sample is induced to remanence magnetization by a saturating magnetic field of 1 kOe applied along the film plane.

The anisotropic magnetoresistance measurements result in a feasible and reliable method to determine the orientation of magnetization in the FeGa thin film. As a matter of fact, *R* varies as a function of the angle between the direction of magnetization and the direction of the electric current (*I*) flowing in the magnetic layer.^[^ ^64^ ^]^ It decreases as the magnetization turns away from the electrical current, reaching a minimum value when they are in a perpendicular configuration and a maximum value when they are in a parallel configuration.

This method succeeds where other ones fail and also does not require expensive and cumbersome instruments, bridging the gap between fundamental research work and real applications.^[^ ^64^ ^]^

## Conflict of Interest

The authors declare no conflict of interest.

## Supporting information



Supporting Information

Supporting Information

## Data Availability

The data that support the findings of this study are available from the corresponding author upon reasonable request.
