# Supplementary material for: Light‐Controlled Magnetic Properties: An Energy‐Efficient Opto‐Mechanical Control over Magnetic Films by Liquid Crystalline Networks
Source: Adv Sci (Weinh). 2024 Oct 7;11(47):2408273. doi: 10.1002/advs.202408273 (PMC11653635; doi:10.1002/advs.202408273)
Supplement: Supplementary file 1 — Supporting Information [file ADVS-11-2408273-s001.pdf]

ADVANCED SCIENCE
Open Access

Supporting Information

for *Adv. Sci.*, DOI 10.1002/advs.202408273

Light-Controlled Magnetic Properties: An Energy-Efficient Opto-Mechanical Control over Magnetic Films by Liquid Crystalline Networks

Gabriele Barrera, Daniele Martella*, Federica Celegato, Neri Fuochi, Marco Coïsson, Camilla Parmeggiani, Diederik S. Wiersma and Paola Tiberto*

Supporting Information

Light-Controlled Magnetic Properties: an Energy-Efficient Opto-mechanical control over magnetic films by Liquid Crystalline Networks

Gabriele Barrera$, Daniele Martella*$, Federica Celegato, Neri Fuochi, Marco Coïsson, Camilla Parmeggiani, Diederik. S. Wiersma, Paola Tiberto*

$ *These authors contributed equally*

Additional experimental methods

Preparation of the monomer mixture. The monomer mixture was prepared with molecules depicted in Figure S1. It contained the monoacrylate LC mesogen **1** (C6BP, 40 % mol/mol), the diacrylate LC mesogen **2** (RM82, 54 % mol/mol), the azobenzene dye **3** (5 % mol/mol) and the photoinitiator **4** (Irgacure 819, 1% mol/mol). The molecules were mixed with 1 mL of dichloromethane and then, the solvent was removed under reduced pressure.

Preparation of the LC cell. A cell composed by two microscope glasses was used to control the actuator thickness and its molecular alignment. First, the glasses were washed with isopropanol and water then, spin-coated with a polyvinyl alcohol (PVA) solution (1% in water) and mechanically rubbed unidirectionally with a velvet cloth. The PVA layer allows to obtain a planar alignment of the mesogens (with molecules that are parallel to the glass surface) while the rubbing is useful to determine the director alignment. Using this treatment on both glasses of the cell is an efficient way to obtain a monodomain homogeneous planar alignment in nematic mixture. Glass spheres (diameter 50 μm) were used as spacers in between the glasses to control the thickness of the final film.

Preparation of the photoresponsive actuators. The monomer mixture was melted in the isotropic phase and infiltrated at 100 °C by capillary action in the LC cell on a hotstage.a After the infiltration, LC cells were cooled down at 45 °C (to obtain the homogeneous planar alignment of the molecules in the nematic phase) and photopolymerized by irradiation with a blue lamp (ThorLabs M450LP1, λ = 450 nm, I: 13.75 mW/cm^2). During the irradiation, the growth of the polymeric chain and their crosslinking occur simultaneously, to afford the final material in a one-step reaction. LC cells were then immersed in water in order to facilitate the

detachment from the glasses and the LCN film was mechanically removed with the aid of a razor blade.

Material characterization. Phase transition temperatures of mixtures were measured by DSC TA Instruments Calorimeter Q-2000 (TA-Instruments, Milan, Italy) in a nitrogen atmosphere (heating and cooling rate: 10 °C/min). Polarized optical microscopy (POM) was performed using a Zeiss Axiolab 5 polarized microscope equipped with the Axiocam 208 color and a Linkam PE120 Peltier Systems to control the sample temperature.

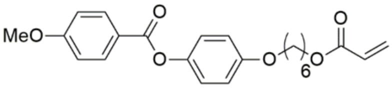

1 (C6BP)

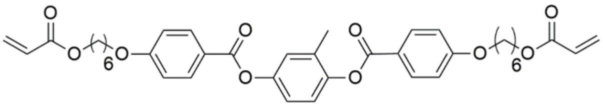

2 (RM82)

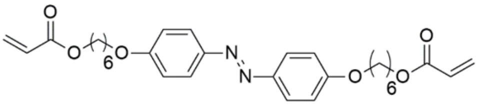

3

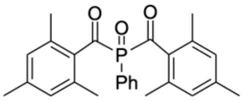

4 (Irgacure 819)

Figure S1. Molecular structure of the monomers used.

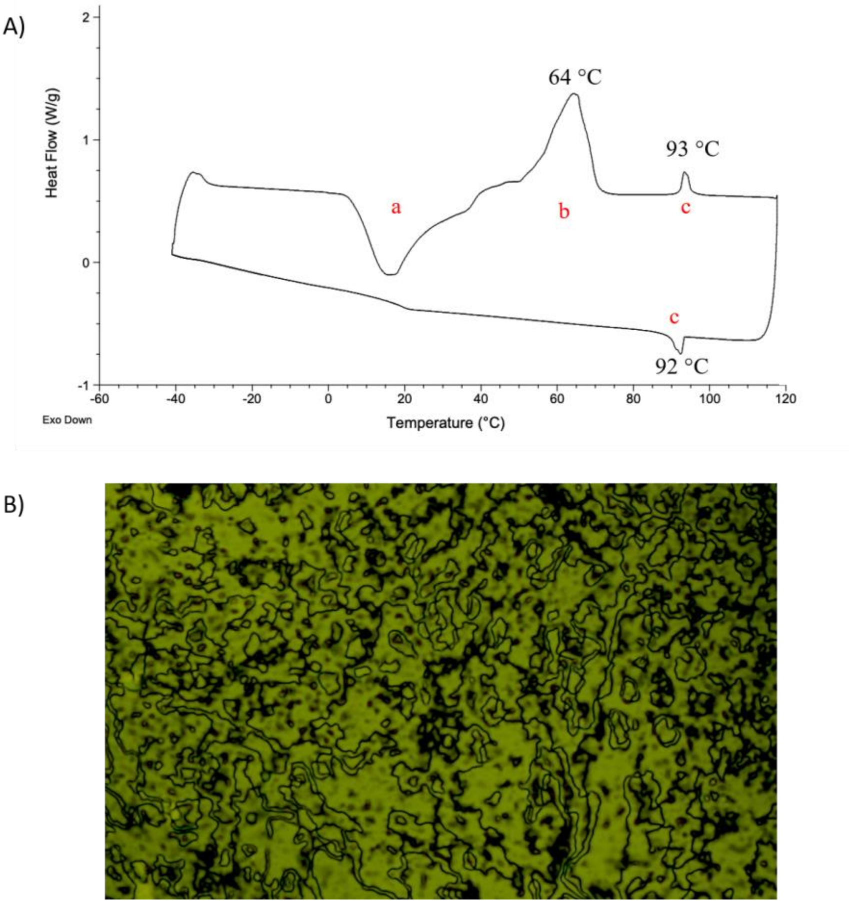

Figure S2. Mesomorphic properties of the LC monomer mixture. A) DSC trace of the second heating-cooling cycle of the mixture (10 °C/min); The analysis shows a cold crystallization (a), the melting form the crystal to the LC phase (b) and the LC to isotropic phase transition (c); B) Representative POM image of the LC phase. The LC phase has been assigned as nematic thanks to the observation of typical Schlieren texture (with simultaneous presence of 2-arm and 4-arm defects).

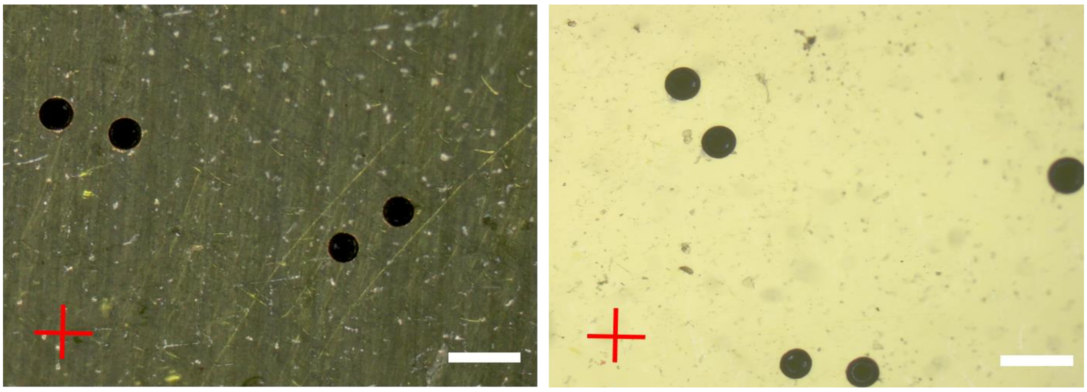

Figure S3. POM images of a LCN film. Image of the film with a nematic director parallel to one polarizer (left) and the same but rotating the sample of 45° (right). The images demonstrate a transmittance change under sample rotation with maximum difference every 45° (as expected for a homogeneous planar alignment); scale bars (in white): 200 μm, the red lines show the linear polarizer and analyzer directions.

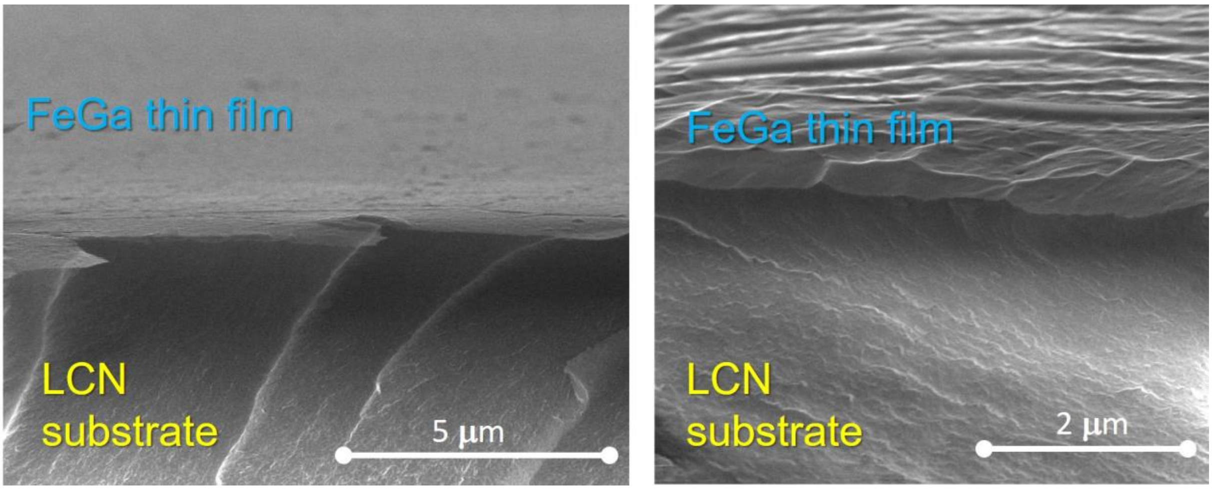

Figure S4. SEM images of the cross section of FeGa/LCN composite material.

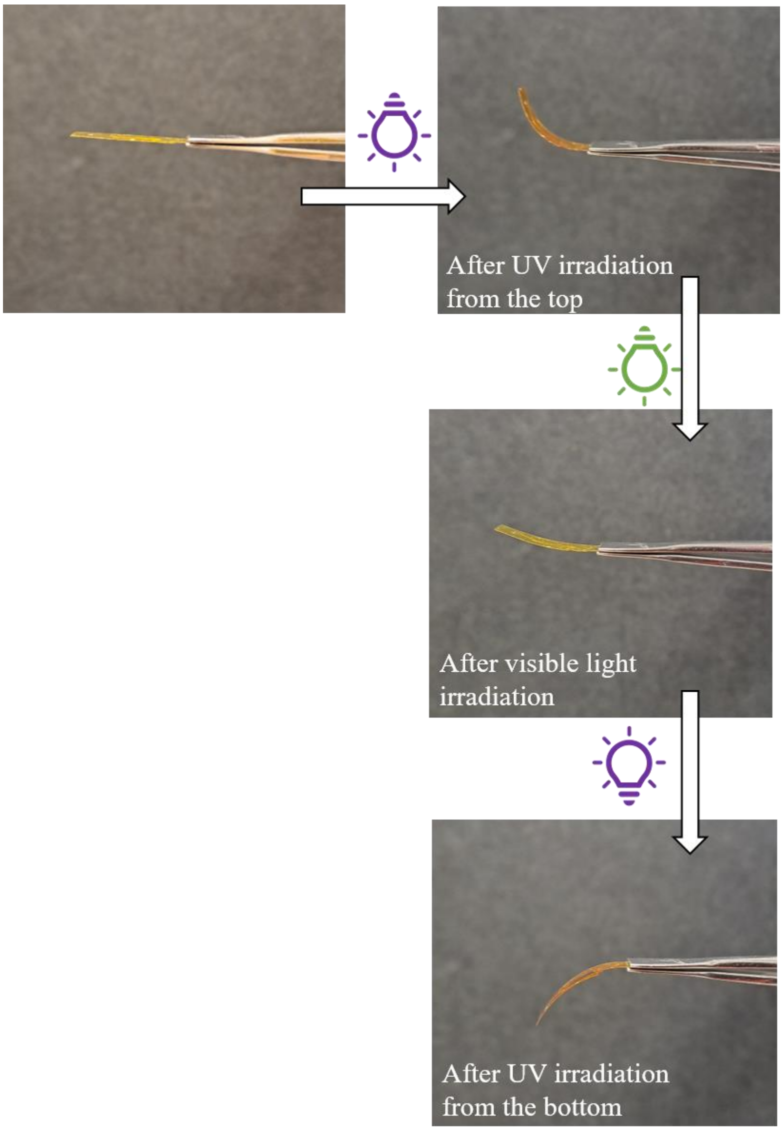

Figure S5. Deformation of a LCN stripe irradiated by different sources. In the sequence of images, the stripe has been irradiated in sequence with: UV light from the top (causing an upward bending), green light (restoring the flat shape) and UV light from the bottom (causing a downward bending).

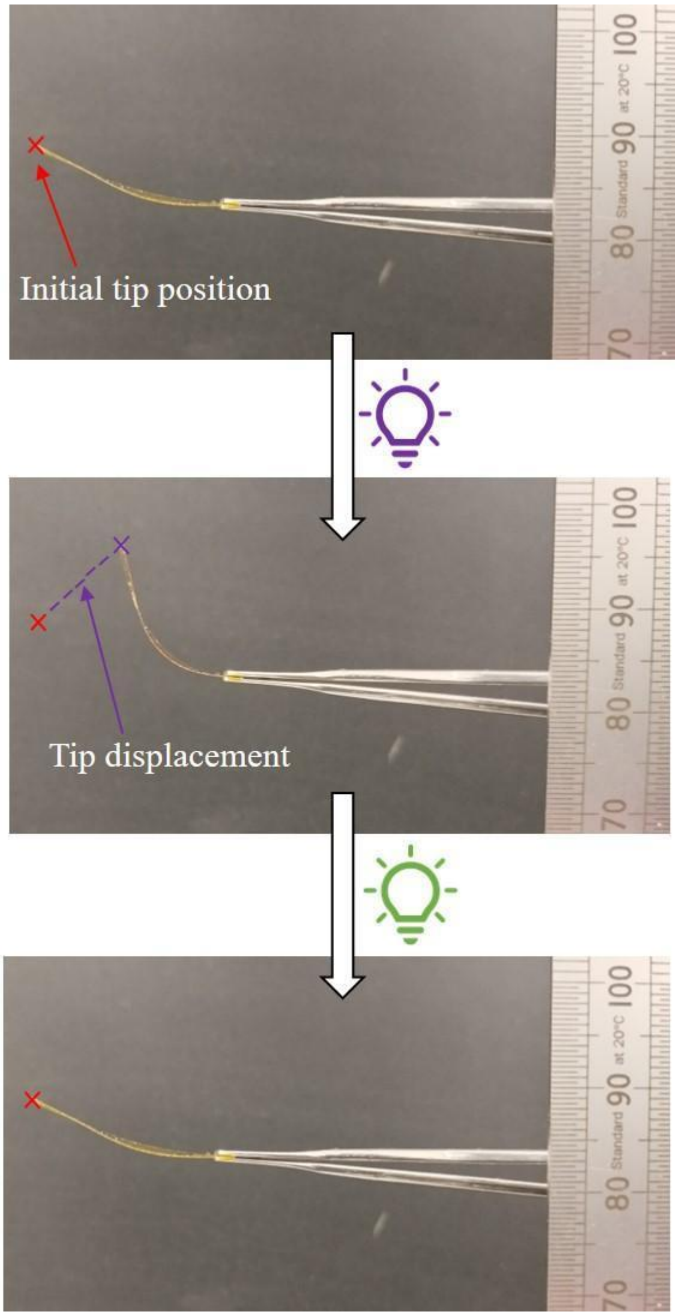

Figure S6. Reversibility of the light induced deformation in a LCN coupled with a metallic layer. In this example, the sequence of pictures shows the initial tip position, its bending after UV exposure (60 seconds of irradiation) and the recovery of the starting position after green light exposure (60 seconds of irradiation).

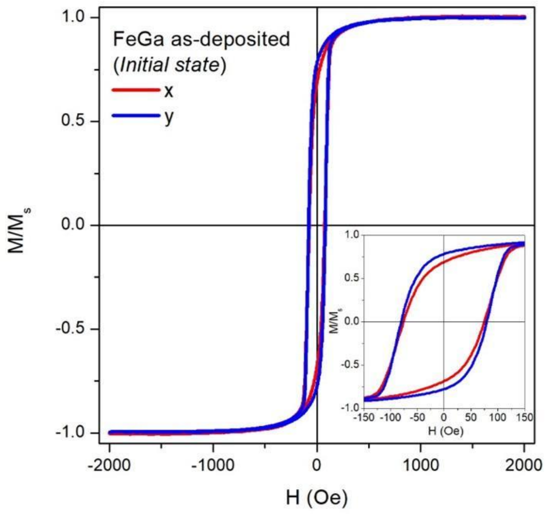

Figure S7: magnetic properties of the pristine FeGa/LCN composite. Room-temperature hysteresis loops of FeGa thin film as-deposited on the photoresponsive LCN substrate applying a magnetic field *H* along both directions in the film plane (labeled *x* and *y*).

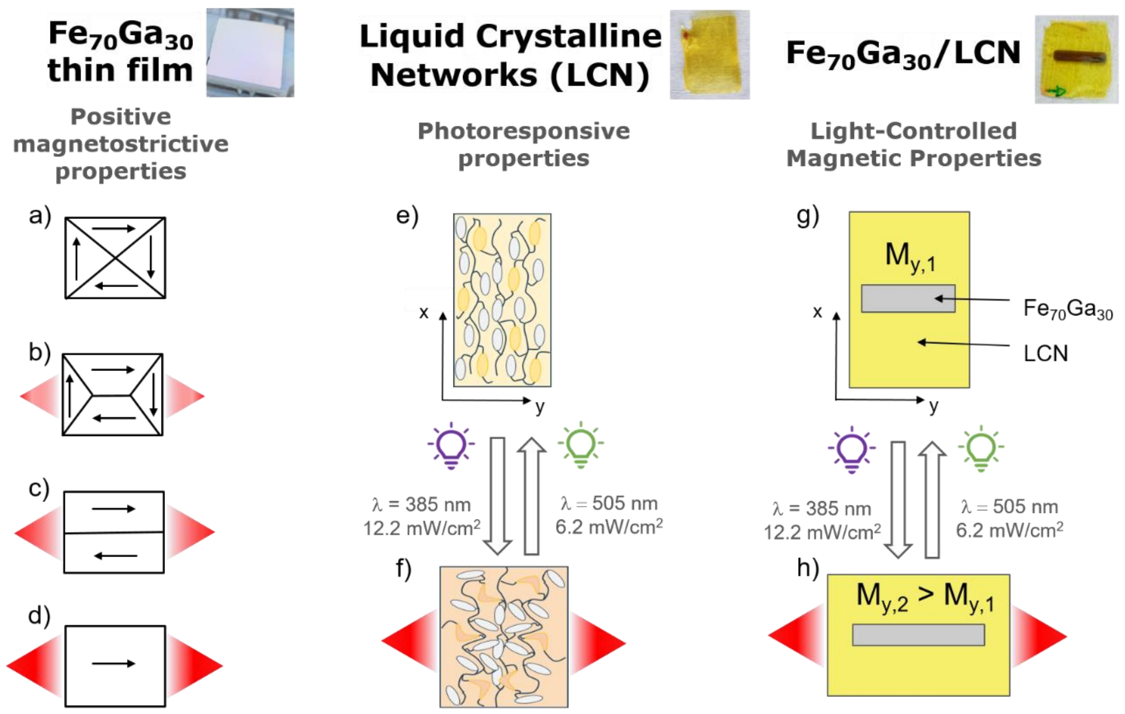

Figure S8: detailed sketch of the functional properties of the materials constituting the photoresponsive FeGa/LCN sample. The $Fe_{70}Ga_{30}$ alloy is a well-known positive magnetostrictive material that exhibits a unique bi-directional coupling between magnetic and mechanical energy. It either responds to an applied magnetic field by exhibiting deformation or

converts external mechanical stress into measurable change in magnetic properties. This latter functionality (called inverse magnetostriction of Villari effect), exploited in the present study, is shown in the left hand side of Figure S8, where the applied mechanical stress (red arrows) alters the domain structure of the FeGa material and creates a new source of magnetic anisotropy. In particular, panel a) represents an ideal small portion of the FeGa thin, comprising four-domains with no preferred orientation due to the polycrystalline structure of the material. The application of a small tensile stress to the sample, as in panel b), induces a movement of the domain walls such that the volume of the domains magnetized perpendicular to the stress axis decreases. These domains are progressively reduced by increasing the applied stress until they disappear completely, resulting in domain structure typical of a uniaxial magnetic anisotropy, see panel c). The transition from panel c) to the full magnetic saturation shown in panel d) can be achieved by applying a small magnetic field that induces wall motion. The strength of this field is less than that required for the four-domain configuration shown in panel a) in the absence of applied stress. As a result, the applied stress strongly affects the magnetic properties of the magnetostrictive material.

Liquid Crystalline Network (LCN) is a material in which mesogenic molecules are chemically crosslinked into polymeric networks and aligned in parallel along a common direction (nematic director), as shown in panel e). By adding an azobenzene crosslinker within LCN, a light irradiation with UV wavelength is exploited to modify the liquid crystalline order, taking advantage of the dye isomerisation from *trans* to *cis* state. As a direct consequence, a macroscopic deformation of the LCN stripe occurs, as shown in panel f). This shape-changing is reversible by using a green light. In the reference system chosen in this work, compression along *x*-direction and elongation along *y*-direction are observed.

The combination of the magnetostrictive $Fe_{70}Ga_{30}$ thin film with LCN substrate, panel g), leads to the realization of a smart composite system, whose shape and magnetic properties are finely tuned by light. UV irradiation of the LCN substrate activates its deformation which is transferred to the magnetostrictive FeGa thin film as a complex mechanical shear stress, thus modifying its magnetic state (M), panel h). The deformation and the magnetic state are maintained even after the light is switched off. In addition, the green light promotes the reversal molecular reconfiguration that restores the pristine magnetic state.

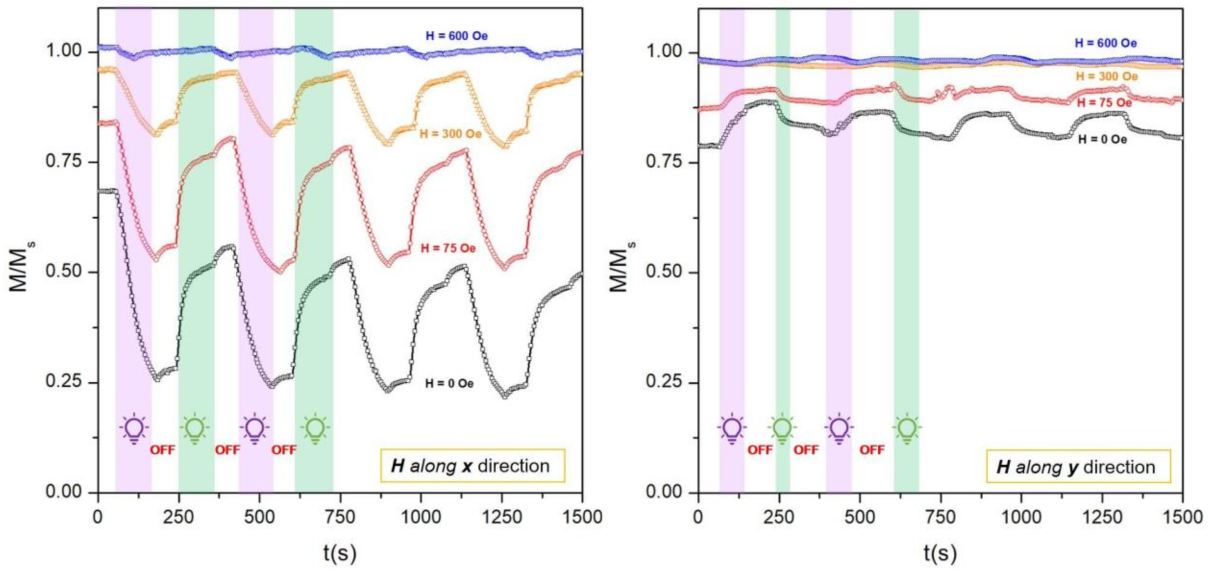

Figure S9: modulation of normalized magnetization by subsequent application of UV and green light for selected values of constant magnetic field applied along the *x*-direction (right panel) and the *y*-direction (left panel).

Figure 2D of the main text shows how the magnetic properties of the FeGa thin film are adjusted without the need for magnetic fields (H = 0). Only the opto-mechanical effect is used by subsequent applications of UV and green light. The same curves are also reported in Figure S9 as black lines.

Furthermore, this M/M_s modulation can be tuned in intensity by applying a constant magnetic field, as shown in Figure S9. In particular, for the same duration of light irradiation, a progressive decrease in the intensity of the M/M_s variation is observed as the magnetic field magnitude increases.

This effect is closely related to the competitive action in orienting the magnetization in the FeGa film of the applied magnetic field and the uniaxial stress anisotropy induced by the opto-mechanical effect. The greater the H strength in orienting the magnetization along its own direction, the less the ability of the uniaxial stress anisotropy to orient the magnetization along its own easy axis. H value above 600 Oe brings the FeGa thin film close to magnetic saturation, completely inhibiting the effect of stress anisotropy.

A greater effect is observed when applying the magnetic field along the *x*-direction (i.e. H and the easy axis are parallel) than in the *y*-direction (i.e. H and the easy axis are perpendicular).

As a matter of fact, the magnetic field can be used as an additional tool to finely control or completely inhibit the variation of magnetization induced by opto-mechanical effect.

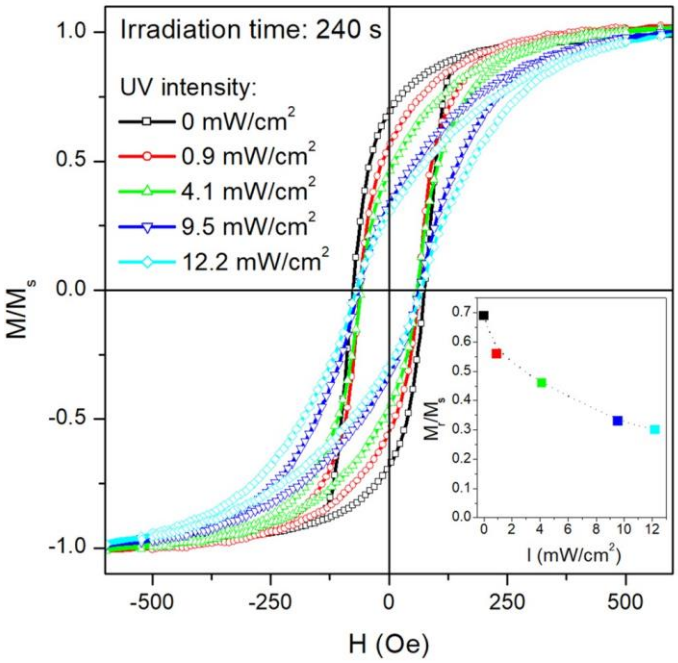

Figure S10: Light-induced modulation of the FeGa/LCN magnetic properties as a function of UV-light source power. Inset: evolution of the M_r/M_s value as a function of light source power. The irradiation was kept fixed at 240s and the measurement was performed along the *x*-direction.

Prior to each magnetic measurement of the hysteresis cycle, the sample was irradiated with UV light for 240 s at selected intensity. After the magnetic measurement, the sample was irradiated with green light for 700 s at maximum intensity to restore the sample to its initial state and then continued with the next UV irradiation at higher power.

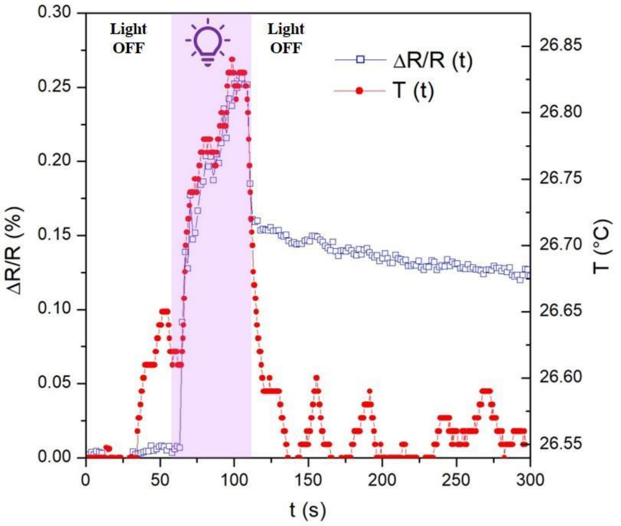

Figure S11: Correlation between resistivity behavior and temperature variation in the FeGa/LCN sample.

The peaks in the Δ*R/R*(t) curves, which occur when the light is switched on (see Figure 3 in the main text), are related to a temperature change in the FeGa thin film induced by light irradiation. To demonstrate this, the temperature of the FeGa thin film was measured using a fiber-optic thermometer placed on the film surface during UV irradiation. Simultaneously, resistance was measured by an electrical current along the *y*-direction.
Figure S11 presents the *ΔR/R*(t) and *T*(t) curves. Upon activation with UV light, the FeGa thin film undergoes a concurrent rise in resistance and temperature. Once the light is turned off, both curves decrease: while the temperature rapidly returns to its initial value, the resistance plateaus at a higher level that is related to the stress induced by the LCN substrate.

While the superimposed *ΔR/R*(t) and *T*(t) curves provide a qualitative understanding, a first-order quantitative analysis is provided using the following equation that relates electrical resistance to temperature:

$$(R(\mathrm{t}) - R_0)/R_0 = \alpha(T-T_0),$$

where R_0 and T_0 are the resistance and temperature of FeGa thin film at t = 0 s, respectively, and α is the temperature coefficient of resistance (TCR). As first approximation, the TCR value of iron ($\alpha = 0.005671\ \mathrm{K}^{-1}$) is used.
Figure S11 shows a Δ*T* of approximately 0.31 °C at the end of UV irradiation, resulting in an expected increase in Δ*R/R* of about 0.18%. This estimation is consistent and of the same order of magnitude with the experimentally Δ*R/R* value shown in Figure S11 and in all the measurements reported in Figure 3.

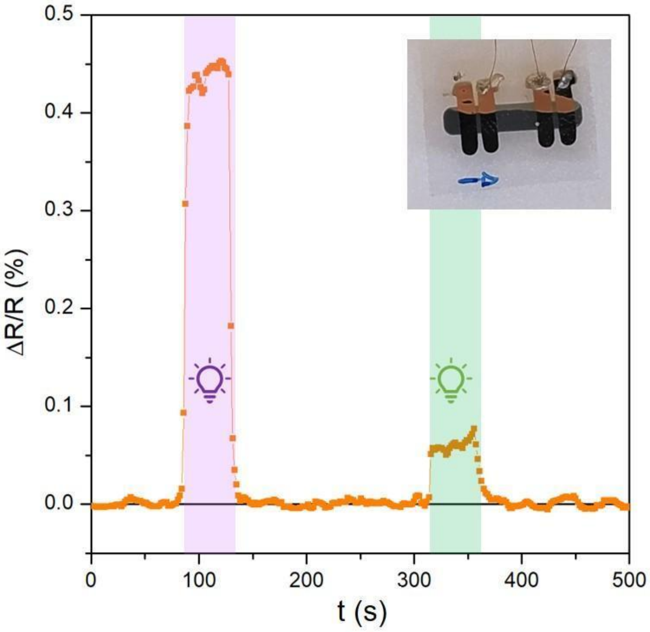

Figure S12: Δ*R/R* measurements as a function of time and light irradiation in FeGa thin film on LCN substrate without photosensitive molecules.

To demonstrate that the ability to fix selected Δ*R/R* values by light irradiation is solely due to the stress induced by the cis-trans dye isomerisation, magnetoresistive measurement was performed on a FeGa/LCN sample without photosensitive molecules in the polymer matrix. This sample is completely transparent (see inset of Figure S12), without the yellow tinge associated with the presence of the dye (refer to Figure 1 in the main text).

As a result, the Δ*R/R(t)* curve returns to its initial value after the light (both UV and green) is switched off. Only the increase due to the thermal effect is visible when the irradiation is active, see Figure S12.

Therefore, irradiation without dye in the polymer matrix cannot set any Δ*R/R* value other than the initial value. (The higher value obtained for the Δ*R/R*(t) curve during the irradiation compared to the results shown in Figure 3 is due to the fact that no fan was used to improve the heat exchange in this experiment).

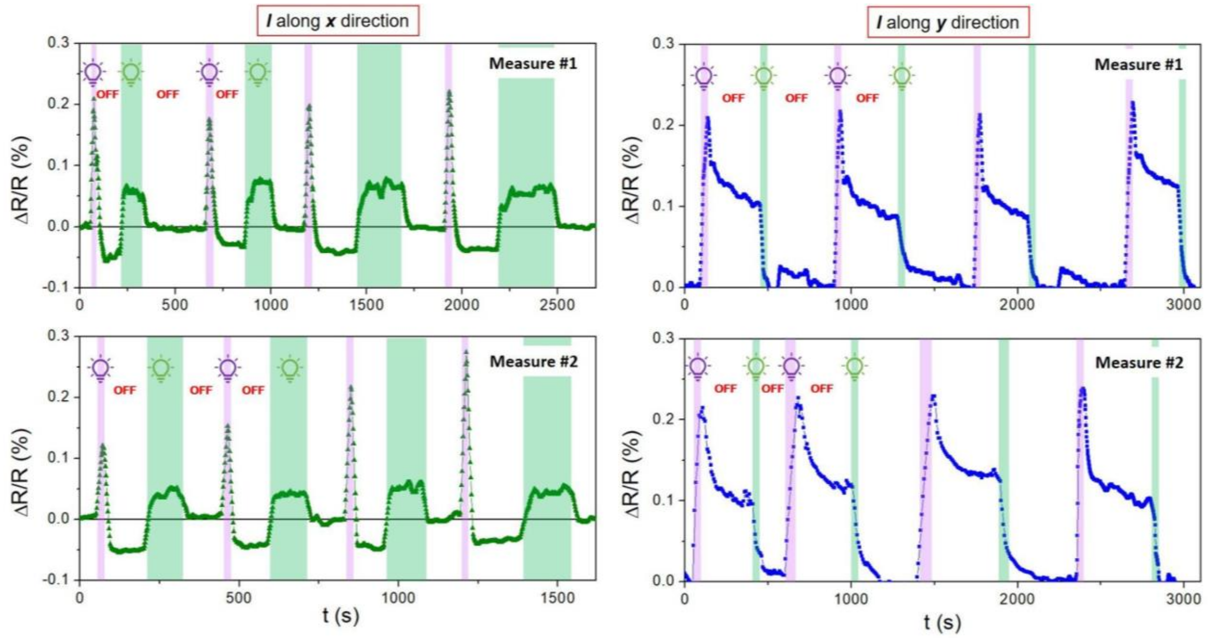

Figure S13: Comparison of two separate sets of measurements on the modulation of the Δ*R/R* value by light irradiation to study the long-term stability of the FeGa/LCN device. Measurements #1 and #2 was performed two months apart. The same samples are used and both electric current configurations are investigated. (The measures #1 are the same reported in Figure 3B and 3C)

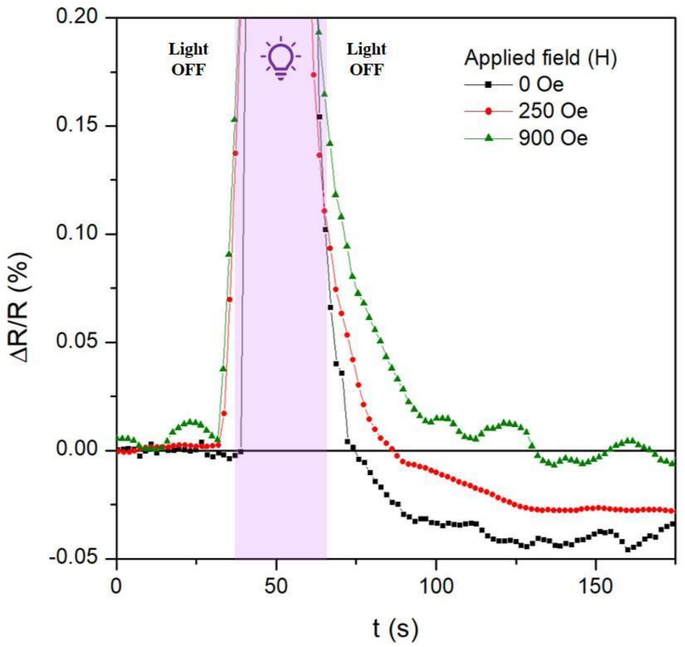

Figure S14: Effect of a constant external magnetic field on the modulation of the Δ*R/R* value induced by UV light

Synthesis of the azobenzene crosslinker 3

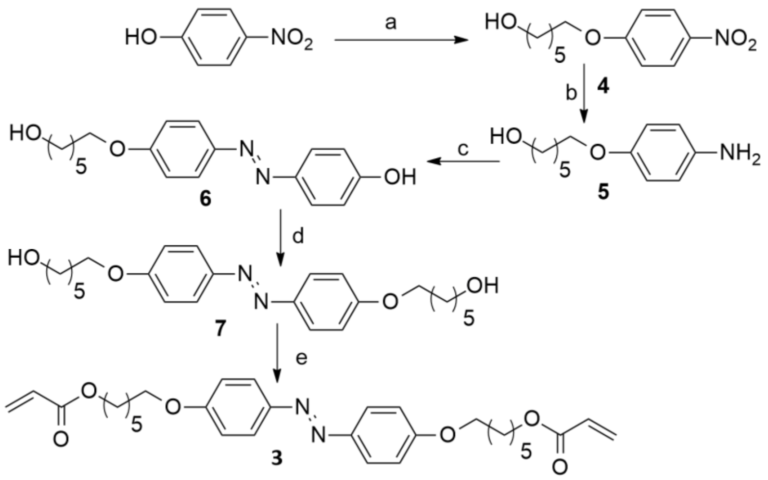

Figure S15. Synthesis of azobenzene crosslinker 3. Reagents and conditions: a) 6-chloro-1-hexanol, K_2CO_3, DMF, 100 °C, 18 h, 82%; b) MeOH, H_2, Pd/C, rt, 48 h, 98%; c) $NaNO_2$, H_20, HCl 37%, 0 °C, 15’, then addition to the phenol in NaOH aqueous solution, 0 °C, 1 h’, 85%; d) 6-chloro-1-hexanol, K_2CO_3, DMF, 100 °C, 18 h, 91%; e) acryloyl chloride, CH_2Cl_2, TEA, rt, 4 h, 67%.

6-(4-nitrophenoxy)hexan-1-ol (**4**). 4-nitrophenol (1 g, 7.19 mmol) and 6-chloro-1-hexanol (1.12 g, 8.63 mmol) were dissolved into a mixture of K_2CO_3 (1.19 g, 8.63 mmol) in DMF (5 mL). After reaction at 100 °C for 18 h, water (30 mL) was added to give a precipitate which was filtered and washed thoroughly with water affording the product in 82% yield (1.42 g, 5.93 mmol) as a yellow solid.^{1}H-NMR (300 MHz, $CDCl_3$) δ 8.21-8.26 (m, 2H, Ar), 6.96-6.90 (m, 2H, Ar), 4.04 (t, J = 6.4 Hz, 2H, ArOCH_2A), 3.65 (t, J = 6.5 Hz, 2H, ACH_2OH), 1.87-1.78 (m, 2H, aliphatic -CH_2-), 1.64-1.42 (m, 6H, aliphatic -CH_2-) ppm.

6-(4-aminophenoxy)hexan-1-ol (**5**). A solution of **4** (1.42 g, 5.93 mmol) in MeOH (30 mL) was hydrogenated in the presence of 10% Pd/C (150 mg) for 48 h. The catalyst was filtered off, washed with methanol and evaporated to give the product with a 98% yield (1.22 g, 5.83 mmol) as a brown solid. ^{1}H-NMR (300 MHz, $CDCl_3$) δ 6.76-6.61 (m, 4H, Ar), 3.88 (t, J = 6.5 Hz, 2H, ArOCH_2A), 3.65 (t, J = 6.3 Hz, 2H, ACH_2OH), 1.79-1.72 (m, 2H, aliphatic -CH_2-), 1.63-1.38 (m, 6H, aliphatic -CH_2-) ppm.

4-((E)-{4-[(6-hydroxyhexyl)oxy]phenyl}diazenyl)phenol (**6**). A solution of **5** (793 mg, 3.79 mmol) in 7 mL of water and 3.5 mL of 37% HCl was stirred at room temperature for 18 h. Then the mixture was cooled at 0 °C, a solution of $NaNO_2$ (295 mg, 4.28 mmol) in water (3 mL) was added dropwise and the mixture stirred for 15 min. Phenol (357 mg, 3.79 mmol) and NaOH

(1.2 g) were placed in another flask, 7 mL of water were added and this solution was kept below 5 °C. Afterwards, the first solution of the diazonium salt was dropped slowly and after 1 h the azobenzene derivative was precipitated using 1 M HCl solution. The precipitate was filtered, washed with water and dried. The product was obtained in 85% yield (1.01 g, 3.21 mmol) and used in the next step without other purification. ^{1}H-NMR (300 MHz, $CDCl_3$) δ 7.88-7.80 (m, 4H, Ar), 7.00-6.91 (m, 4H, Ar), 4.04 (t, *J* = 6.5 Hz, 2H, ArOCH_2A), 3.68 (t, *J* = 6.5 Hz, 2H, ACH_2OH), 1.88-1.76 (m, 2H, aliphatic -CH_2-), 1.64-1.47 (m, 6H, aliphatic -CH_2-) ppm.

6,6'-[(E)-diazene-1,2-diylbis(4,1-phenyleneoxy)]dihexan-1-ol (**7**). **6** (1.29 g, 4.10 mmol) and 6-chloro-1-hexanol (672 mg, 4.92 mmol) were dissolved into a mixture of K_2CO_3 (679 mg, 4.92 mmol) in DMF (10 mL). After reaction at 100 °C for 18 h, water (60 mL) was added to give a precipitate which was filtrated and washed thoroughly with water affording the product in 91% yield (1.55 g, 3.74 mmol). ^{1}H-NMR (300 MHz, $CDCl_3$) δ 7.88-7.83 (m, 4H, Ar), 7.00-6.96 (m, 4H, Ar), 4.04 (t, *J* = 6.3 Hz, 4H, ArOCH_2A), 3.68 (t, *J* = 6.1 Hz, 4H, ACH_2OH,), 1.88-1.78 (m, 8H, aliphatic -CH_2-), 1.66-1.45 (m, 8H, aliphatic -CH_2-) ppm.

(E)-diazene-1,2-diylbis(4,1-phenyleneoxyhexane-6,1-diyl) bisacrylate (**3**). To a solution of **7** (1.55 g, 3.74 mmol) in dry CH_2Cl_2 (100 mL), TEA (2.26 g, 22.374 mmol) and acryloyl chloride (810 mg, 8.95 mmol) were added and then the mixture was stirred at room temperature under N_2 atmosphere for 4 h. The solution was washed with water (3x100 mL) and the organic layers dried over Na_2SO_4, filtered and evaporated under. The crude product was recrystallized from methanol affording the desired product in 67% yield (1.31 g, 2.51 mmol) as a yellow solid. ^{1}H-NMR (300 MHz, $CDCl_3$) δ 7.90-7.85(m, 4H, Ar), 7.00-6.96 (m,4H, Ar), 6.41 (dd, *J* = 17.2, 1.6 Hz, 2H, CH=CH_2), 6.12 (dd, *J* = 17.2, 10.2 Hz, 2H, C*H*=CH_2), 5.80 (dd, *J* = 10.2, 1.6 Hz, 2H, CH=CH_2), 4.18 (t, *J*= 6.6 Hz, 4H, ArOCH_2A), 4.04 (t, *J* = 6.3 Hz, 4H,ACH_2OCO), 1.87-1.66 (m, 8H, aliphatic -CH_2-), 1.52-1.46 (m, 8H, aliphatic -CH_2-) ppm.

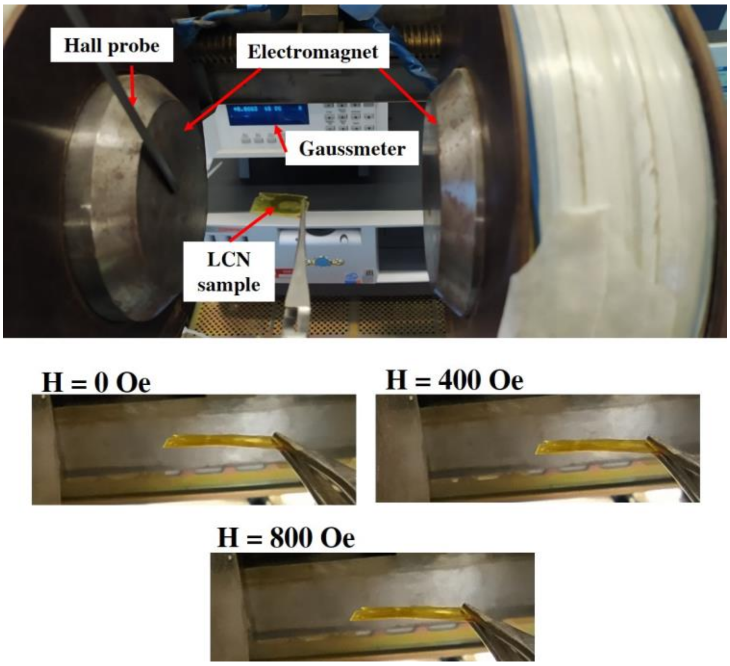

Figure S16: Experimental setup to test the effect of magnetic field on a LCN polymer substrate. Selected field intensities were applied and no shape change of the LCN strip was observed, i.e. the magnetic field does not affect the orientation of the mesogens.

Movie S1. Light induced deformation in a LCN with a magnetic skin. The laminated LCN strip has been irradiated first with UV light and then with visible one.
